# Automatic Classification of Specific Melanocytic Lesions Using Artificial Intelligence

**DOI:** 10.1155/2016/8934242

**Published:** 2016-01-17

**Authors:** Joanna Jaworek-Korjakowska, Paweł Kłeczek

**Affiliations:** Department of Automatics and Biomedical Engineering, AGH University of Science and Technology, Aleja Mickiewicza 30, 30-059 Krakow, Poland

## Abstract

*Background*. Given its propensity to metastasize, and lack of effective therapies for most patients with advanced disease, early detection of melanoma is a clinical imperative. Different computer-aided diagnosis (CAD) systems have been proposed to increase the specificity and sensitivity of melanoma detection. Although such computer programs are developed for different diagnostic algorithms, to the best of our knowledge, a system to classify different melanocytic lesions has not been proposed yet.* Method*. In this research we present a new approach to the classification of melanocytic lesions. This work is focused not only on categorization of skin lesions as benign or malignant but also on specifying the exact type of a skin lesion including melanoma, Clark nevus, Spitz/Reed nevus, and blue nevus. The proposed automatic algorithm contains the following steps: image enhancement, lesion segmentation, feature extraction, and selection as well as classification.* Results*. The algorithm has been tested on 300 dermoscopic images and achieved accuracy of 92% indicating that the proposed approach classified most of the melanocytic lesions correctly.* Conclusions*. A proposed system can not only help to precisely diagnose the type of the skin mole but also decrease the amount of biopsies and reduce the morbidity related to skin lesion excision.

## 1. Introduction

Body organs are not all internal like the heart, brain, or liver. There is one we wear on the outside which protects us from extremes of temperature, damaging sunlight, and harmful chemicals. Skin is our largest organ which in adolescence covers about 2 square meters and weights 3.6 kilograms [1]. Like in other parts of the body, skin cells can also grow abnormally, causing cancerous tumors to form. The most popular and widely used method to analyze skin changes is to observe them under a skin surface microscope.

**Figure 1:**
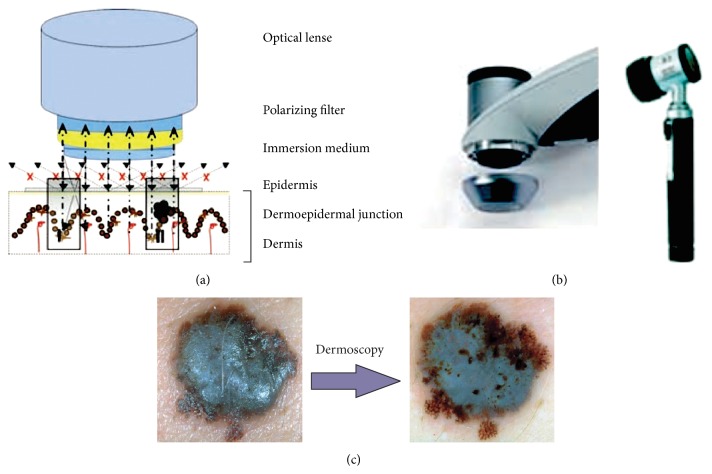
(a) Specific optical system for the pigmented skin lesion examination. The mole is typically covered with a liquid (usually oil or alcohol). (b) Dermoscopy is performed with a handheld instrument called a dermatoscope. (c) Dermoscopy enables clinicians to observe border irregularities, colors, and structures within skin lesions that are otherwise not visible to the unaided eye [2, 4].

The analysis of skin with a microscope started in 1663 by Kolhauser who observed vessels of nail matrix. In 1958, after many years of research, the first portable dermatoscope has been produced [2]. Goldman was the first dermatologist to coin the term “dermascopy” and to use the dermatoscope to evaluate pigmented cutaneous lesions. Nowadays the examination of the small moles is possible through a digital epiluminescence microscopy (ELM, also dermoscopy or dermatoscopy) which is a noninvasive,* in vivo* technique that, by employing the optical phenomenon of oil immersion, makes subsurface structures of the skin accessible for examination in an optic magnification between 10 to 40 times [2].  Figure 1 presents comparison between the skin mole observed with the naked eye and with a dermoscope. Dermoscopy enables clinicians to observe global and local structures very precisely and thus provides the additional criteria for the clinical diagnosis of pigmented skin lesion.

This paper is organized in four sections as follows.  Section 1 covers background information on the nature of skin cancer, presents the clinical definition of different melanocytic lesions, and describes the motivation and state of the art.  Section 2 specifies melanocytic lesions classification algorithm, including the following steps: preprocessing, segmentation, feature extraction, feature selection, and classification method. In Section 3, the conducted tests and results are described.  Section 4 closes the paper and highlights future directions.

**Figure 2:**
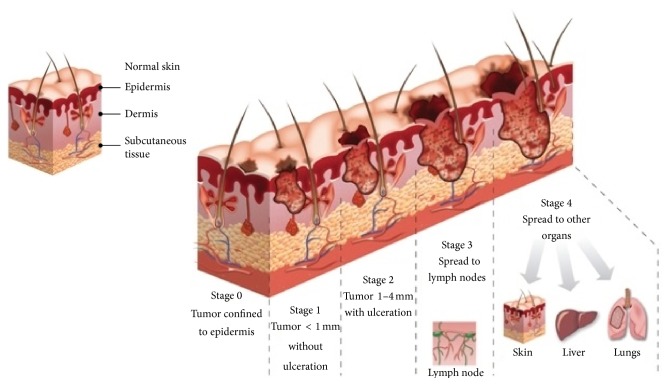
Comparison between healthy skin and skin affected by malignant melanoma. Presentation of five stages in malignant melanoma evolution process [5].

*Background*. Although the two most commonly diagnosed skin cancers are basal cell carcinoma and squamous cell carcinoma, which develop from the nonpigmented cells of the skin, the most aggressive and dangerous is malignant melanoma. Malignant melanoma (Latin:* melanoma malignum*) originates in pigment producing cells called melanocytes and is less common but far more deadly than cancers mentioned above [3]. Melanomas are fast-growing and highly malignant tumors often spreading to nearby lymph nodes, lungs, and brain (Figure 2). Malignant melanoma is likely to become one of the most common malignant tumors in the future, with even a ten times higher incidence rate [2].

Due to the high skin cancer incidence and mortality rates, early diagnosis of melanoma has become an extremely important issue. The progress is visible not only in the primary research but also in the development of sophisticated, more accurate methods of image processing, classification, and computer-aided diagnosis [4].

In this paper we present a new approach to the classification of melanocytic lesions. Most of the research done so far in this area has concentrated on creating new methods to distinguish benign from malignant skin lesions. In our research we go one step further and differentiate melanocytic lesions including Clark nevus, Spitz nevus, blue nevus, and malignant melanoma. In the next part of this paper we will present the clinical importance and motivation to undertake this research. 

*Clinical Importance and Motivation*. A melanocytic nevus (also called a nevocytic nevus or a mole) is a lesion that contains nevus cells (a type of pigment cells called melanocytes). A mole can be either subdermal (under the skin) or a pigmented growth on the skin, formed mostly of melanocytes. The high concentration of the body's pigmenting agent, melanin, is responsible for their dark color. Although melanocytic nevi are very common, their histogenesis is not well understood and still a matter of debate [4]. All we know about the life of melanocytic nevi is based on cross section or cohort studies, because it is still complicated to monitor skin lesions* in vivo* on a cellular level. The majority of moles appear during the first two decades of a person's life. Acquired moles are a form of benign neoplasms, while congenital moles (or congenital nevi) are considered a minor malformation or hamartoma and may be at a higher risk of melanoma [6].

In our research we concentrate on the classification of the most popular melanocytic nevi to the diagnostic categories including Clark nevus, Blue nevus, Spitz/Reed nevus, and malignant melanoma. It is of high importance to diagnose correctly the type of a skin lesion, because the further physicians' orders depend on it and some of the nevi have a higher risk of developing malignant melanoma than other ones. The proposed system can not only help to precisely diagnose the type of the skin mole but also decrease the amount of biopsies and reduce the morbidity related to skin lesion excision. 

**Figure 3:**
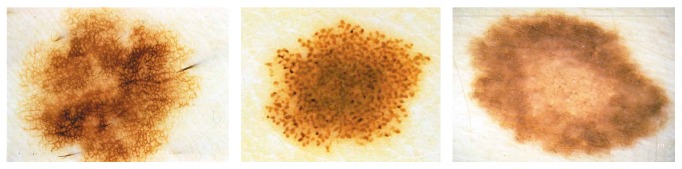
Reticular and globular type of Clark nevus, based on [2].

*Clark Nevus*. Clark nevi have been named in 1978 after Wallace H. Clark, Jr., who described this particular type of nevus by studying numerous melanocytic nevi in patients with concomitant melanomas [2]. Clark nevi are the most common nevi in man, and while these moles are neither contagious nor dangerous, medical experts believe that Clark nevi do present a higher risk of turning into melanoma when compared to more common moles. Clinical, dermoscopic, and histopathologic variants of Clark nevi are protean, and the differentiation of Clark nevi from melanoma* in situ* and early invasive melanomas is the major challenge in the realm of pigmented skin lesions [2]. Clinically, Clark nevi are flat to elevated or even slightly papillated pigmented lesions characterized by various shades of brown coloration and situated on the trunk and extremities that are usually called common junctional nevi or common compound nevi (Figure 3). Those who have a number of Clark nevi should pursue a complete skin examination every year. 

**Figure 4:**
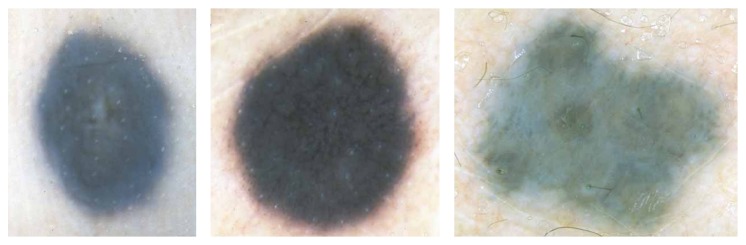
Examples of blue nevus, based on [2].

*Blue Nevus*. According to the original definition by Tieche, blue nevus is a dermal-based, benign melanocytic lesion histopathologically made up by variable proportions of oval/spindle and bipolar, usually heavily pigmented dendritic cells [2, 4, 6]. Clinically, blue nevi appear as relatively regular, sharply circumscribed with a uniform blue to gray-blue or sometimes even gray-black pigmentation. Dermoscopically, blue nevi exhibit a homogeneous pattern with complete absence of local features, such as pigment network structures, brown globules, or black dots within the diffuse homogenous pigmentation (Figure 4). This absence of local features and the presence of a well-defined border, usually without streaks, are criteria to differentiate blue nevus from melanoma, in many cases with a high degree of certainty. As blue nevi are harmless no treatment is needed. 

**Figure 5:**
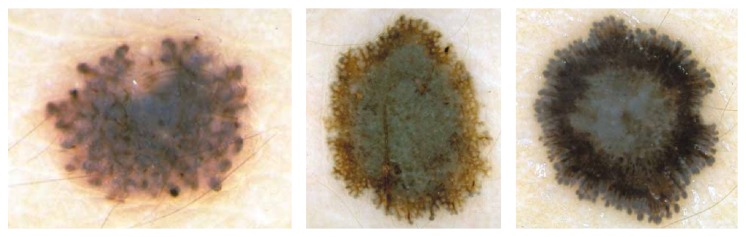
Examples of Spitz/Reed nevus, based on [2].

*Spitz/Reed Nevus*. Spitz/Reed nevi are typically small, well-circumscribed, reddish papules, larger reddish plaques, but also verrucous plaques with variegated colors and may be up to one or two centimeters in diameter. Dermoscopically, about 50% of pigmented Spitz nevi show a symmetric appearance and a characteristic starburst pattern or a globular pattern with a regular, discrete blue pigmentation in the center and a characteristic rim of large brown globules at the periphery (Figure 5). Spitz nevi are well-known simulators of cutaneous melanoma from a clinical, dermoscopic, and histopathologic point of view [2]. 

*Malignant Melanoma*. Malignant melanoma is the most aggressive type of skin cancer. It is due to the uncontrolled growth of melanocytes. The first sign of melanoma is usually an unusual looking mole or spot. Melanoma may be detected at an early stage when melanocytic lesions are only a few millimeters in diameter, but they also may grow up to several centimeters in diameter before being diagnosed.

**Figure 6:**
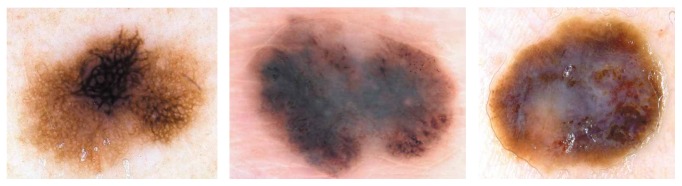
Examples of malignant melanoma, based on [2].

Clinically, a melanoma can have a variety of colors including white, brown, black, blue, red, or even light grey. Melanomas in early stage are usually small, more or less irregularly shaped and outlined macules, or slightly elevated plaques with pigmentation that varies from pink to dark brown or black. Invasive melanomas are, as a rule, papular or nodular, often ulcerated, and characteristically exhibit shades of brown and black but also foci of red, white, or blue coloration. Dermoscopic features describing melanoma contain multicomponent pattern, irregular dots/globules, atypical pigment network, irregular streaks, irregular pigmentation, regression structures, and blue whitish veil (Figure 6). A suspected melanoma should be surgically removed with a 2-3 mm margin (excision biopsy) and sent to a pathology laboratory for a microscopic examination [2]. 

*Related Works*. During patient examinations researchers observe that young inexperienced dermatologists and family physicians have huge difficulties in the correct visual assessment of skin lesions. As stated in [7, 8] only experts have arrived at 90% sensitivity and 59% specificity in skin lesion diagnosis, while for unexperienced physicians these figures show a significant drop till around 62-63% for general practitioners [9]. Due to these obstacles, researchers try to implement and build computer-aided diagnosis (CAD) systems for automated diagnosis of melanoma to increase the specificity and sensitivity and to simplify the assessment of skin moles. Two reviews on the state of the art of CAD systems for skin lesion diagnosis can be found in [3, 10]. For these systems, the true positive rate ranges between 0.8 and 1.0 and the true negative rate between 0.5 and 0.95. Although such computer programs are developed for different diagnostic algorithms, to the best of our knowledge, a CAD system to classify different melanocytic lesions has not yet been proposed. Based on the review article [3], in Table 1 we present an extended categorization of feature descriptors which are commonly used in the computerized analysis of dermoscopic images.

## 2. Materials and Methods

**Figure 7:**
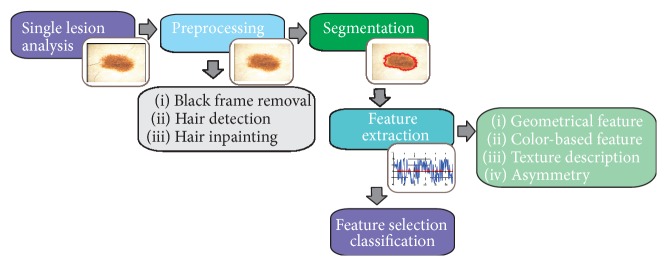
The proposed algorithm for the classification of melanocytic lesions based on dermoscopic color images.

The designed system for the automated diagnosis of melanocytic lesions is a computer-aided diagnosis system which is designed to reproduce the decision of dermatologist based on the dermoscopy images. The proposed methodology of discrimination between malignant melanoma and nevi tumors is shown in Figure 7. The automated system is divided into six main stages, including preprocessing (image enhancement), segmentation, feature extraction, feature selection, classification, and then evaluation. The system enables texture analysis without being limited by selection and detection of structure of interest.

In this section the preprocessing and segmentation steps are described shortly, based on our previous work, while the main stage which is feature extraction, selection, and classification is presented in detail. The application has been implemented using Matlab ver. 2013.

### 2.1. Dermoscopic Image Preprocessing

The main goal of the preprocessing step is to improve the image quality by reducing or even removing the unrelated and surplus parts in the dermoscopic images. Dermoscopic images are inhomogeneous and complex. For dermoscopy images, the preprocessing step is obligatory, because of extraneous artifacts, such as skin lines, air bubbles, and hairs, which appear in virtually every image.

To enhance the image and to reduce the influence of light hairs, air bubble, small pores, shines, and reflections, a median filter is being used.

Among the most necessary artifact rejection steps is hair removal, because hairs may cover parts of the image and make the segmentation and texture analysis impossible. A number of methods have been developed for hair removal in dermoscopic images and they were mostly based on morphological operations and adaptive thresholding [11–13]. A good approach for hair removal is the use of top-hat transformation. The process consists of four steps: converting RGB to grayscale image, applying black top-hat transformation, distinguishing hairs from other local structures, and inpainting.

**Figure 8:**
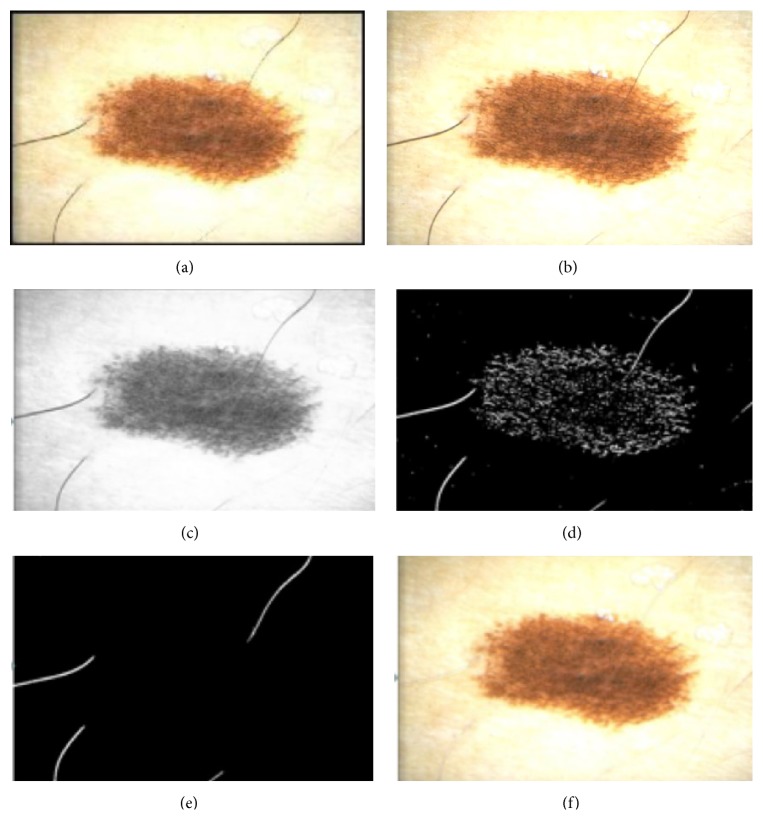
Outcome of the preprocessing step: (a) input image, (b) black frame removal, (c) grayscale conversion, (d) top-hat transform and binarization process, (e) hair distinction from other structures, and (f) inpainting.

The dermoscopic RGB image is being converted into grayscale with the NTSC 1953 standard (Figure 8(c)). Secondly, the black top-hat transform, which is a morphological image processing technique, is used to detect thick, dark hairs. The result of this step is the difference between the closing operation and the input image:

(1) $$\begin{array}{c} T_{w}\left(\;I\;\right)=I\circ b-I, \end{array}$$

where ∘ denotes the closing operation, *I* is the grayscale input image, and *b* is a grayscale structuring element [14, 15].

The black top-hat transform returns an image, containing elements that are darker than their surrounding and smaller than the structuring element.

These steps have been precisely described in our work [14, 15].  Figure 8 presents the outcome after each step.

### 2.2. Segmentation Algorithm

The techniques and algorithms available for segmentation of medical structures are specific to application, imaging modality, and type of body part to be studied. For the dermoscopic images, segmentation process is one of the most challenging and crucial processes. This process for dermoscopic images is extremely difficult due to several factors: low contrast between the healthy skin and moles, variegate coloring inside lesions, and irregular borders, as well as different artifacts. Due to the difficulties described above, numerous methods have been implemented and tested [3]. Celebi et al. present in their research [16] the state of the art of segmentation methods and compare them with the statistical region merging as a recent color image segmentation technique based on region growing and merging. Based on the achieved results and conducted experiments, the skin lesion extraction is performed by seeded region-growing algorithm [17], in regard to two aspects. Firstly, during the preprocessing step, the healthy skin becomes homogeneous. Secondly, the whole skin mole is visible in the dermoscopic image and surrounded by healthy skin. It means that the healthy skin surrounds the mole. Region-growing techniques generally give better results in noisy images where edges are extremely difficult to detect.

**Figure 9:**
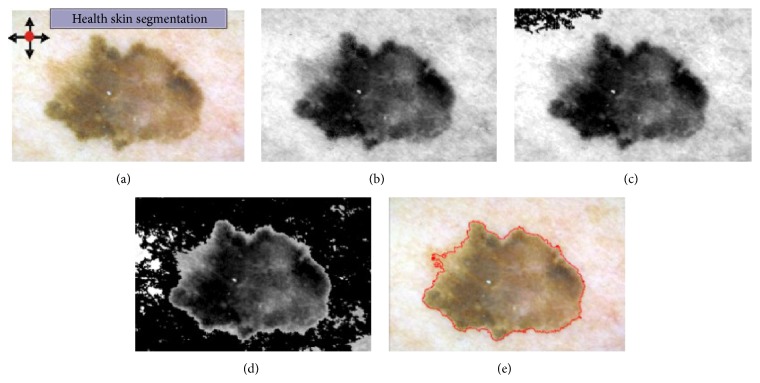
Results of the segmentation process: (a–d) segmentation of the healthy skin with the region-growing algorithm; (e) segmented area.

For the skin lesion segmentation, we take one seed which is located in the left upper corner of the image (Figure 9(a)). The region is iteratively grown by comparing all unallocated neighboring pixels to the region. The region-growing process consists of picking a seed from the set, investigating all 4-connected neighbors of this seed, and merging suitable neighbors to the seed. The seed is then removed and all merged neighbors are added to the seed set. The region-growing process continues until the seed set is empty.

These steps have been precisely described in our work [14]. In Figure 9 we present the results of the segmentation step for several iterations of the region-growing algorithm.

### 2.3. Geometrical Feature Extraction

Geometrical features have been used mainly to describe lesion's outline, as its irregularity usually indicates malignancy. Those features are based mostly on such properties of an object, as area, diameters, or geometric moments. To ensure robustness of the selected features, their formulation does not depend directly on object's perimeter (as in case of raster images it is hard to accurately estimate this quantity). All features are normalized to ensure their scale-, rotation-, and translation-invariance.

To assess lesion's shape, the following features have been extracted: maximal diameter (maximum distance between two arbitrary points), equivalent diameter ($D_{eq}=\sqrt{\left(4/\pi \right)\cdot Area}$), variance of the radial distance distribution, rectangularity (ratio of area of an object to area of its bounding box), elongation (aspect ratio of object's bounding box), eccentricity, Haralick's compactness, and normalized discrete compactness [18–21].

Variance of the radial distance distribution is given by [18]

(2) $$\begin{array}{c} {s_{d}}^{2}=\frac{1}{card\left(B\right)}\frac{\sum_{p\in B}{\left(d\left(p,C\right)-\bar{d}\left(p,C\right)\right)}^{2}}{\bar{d}{\left(p,C\right)}^{2}}, \end{array}$$

where *B* is a set of all pixels constituting perimeter of object *O*, *C* is geometric centroid of *O*, and *d*(*a*, *b*) is the distance between pixels *a* and *b* in a chosen metric. As studied lesions are of circular shape, the Euclidean metric has been chosen.

Eccentricity measures deviation of a conic curve from a circle and (in mathematics) is formulated as ratio of distance between foci of an ellipse to the length of its major axis. In image processing, eccentricity of an object, *O*, is actually value of eccentricity of an ellipse with same second moments as object *O* [19]:

(3) $$\begin{array}{c} \varepsilon =\frac{{\left(m_{02}-m_{20}\right)}^{2}+4m_{11}}{{\left(m_{02}+m_{20}\right)}^{2}}, \end{array}$$

where *m*_*pq*_ denotes an image moment of (*p*, *q*) order. However, such a formulation of eccentricity still serves the purpose of measuring circularity of an object.

Haralick's compactness is a measure for circularity of a digital figure, defined as *μ*_*R*_/*σ*_*R*_, where *R* is a random variable of the distance between the center of the figure to any part of its perimeter [20].

Normalized discrete compactness *C*_*DN*_ measure is based on counting the number of cell sides common to adjacent pixels of object's perimeter, a measure called discrete compactness *C*_*D*_ [21]:

(4) CDN=CD−CDminCDmax−CD=P−2n+2P+4n,CD=4n−P2,  CDmin=n−1,  CDmax=4n−4n2,

where *C*_*D*__min_, *C*_*D*__max_ are, respectively, lower and upper bound of discrete compactness of a shape composed of *n* pixels and *P* is the perimeter of the digital region [21].

### 2.4. Color-Based Features

The analysis of lesion's colors is an important source of information when determining lesion's type, as malignant lesions are characterized by a rich texture [22].

The following color-based features have been extracted: number of colors present within lesion's area (together with information about the presence of two specific colors: white and black), concentricity, centroid distance, and *L* ^*∗*^ *a* ^*∗*^ *b* ^*∗*^ histogram distances [23, 24].

To determine the number of colors and to calculate the concentricity, an image has been converted to CIE *L* ^*∗*^ *a* ^*∗*^ *b* ^*∗*^ color space and then *a* ^*∗*^ and *b* ^*∗*^ color channels have been clustered into four clusters. Three clustering algorithms have been tested: *k*-means clustering, kernel *k*-means clustering, and hierarchical agglomerative clustering [25, 26]. Kernel *k*-means algorithm has been tested using Gaussian kernel for *σ* ∈ {1,2} [26]. In case of hierarchical agglomerative clustering, Ward's minimum variance method based on Euclidean distance between clusters has been applied as the criterion for choosing the pair of clusters to merge at each step [27].

The number of colors has been related to the greatest distance between clusters' centroids (each pair of clusters had been considered) and it has been assumed that to identify two colors as significantly different the distance between them in *a* ^*∗*^ *b* ^*∗*^ space must exceed certain threshold value:

(5) ncolors=max⁡1,1τmax⁡D,D=dEc1,c2:c1,c2∈C∧c1≠c2,

where *C* is a set of clusters' centroids. The threshold value *τ* has been set to *τ* = 12.

It has been assumed that, to state the presence of a certain color within the lesion, the area of biggest cohesive area of that color must be greater than 1% of the area of the whole lesion. Pixels have been recognized as white if *L* ^*∗*^ > 65 and as black if *L* ^*∗*^ < 15, where *L* ^*∗*^ is the value of luminosity channel for the given pixel.

Based on the aforementioned clusterization, a measure called “concentricity” is derived [23]. To compute concentricity of an object, its color clusters are first arranged in ascending order regarding the area of their convex hull (i.e., segment *S*_1_ has the smallest area of convex hull and segment *S*_4_ the greatest), and then a few auxiliary indexes are calculated. The *n*(·) function stands for the number of pixels of the given object.

For segment *S*_1_, the percent area index, PA, is computed [23]:

(6) $$\begin{array}{c} PA=\frac{n\left(CC_{max}\right)}{n\left(S_{1}\right)}, \end{array}$$

where *CC*_max_ is the largest cohesive set of pixels of *S*_1_. The PA describes how well the core is grouped.

Let Hull denote the minimal convex area which includes the entire pixels of *S*_2_. The core inclusion, CI, is then defined as [23]

(7) $$\begin{array}{c} \text{CI}=\frac{n\left(S_{1}\cap \text{Hull}\right)}{n\left(S_{1}\right)}. \end{array}$$

The CI describes how well the second smallest area encircles the core.

Let Core denote the minimal convex area, which includes the entire pixels of *S*_1_. The hull exclusion, HE, is given by [23]

(8) $$\begin{array}{c} \text{HE}=1-\frac{n\left(S_{2}\cap \text{Core}\right)}{n\left(S_{2}\right)}. \end{array}$$

The HE describes how exclusively the hull surrounds the core.

Finally, concentricity is defined as the product of the three variables [23]:

(9) Concentricity=PA×CI×HEConcentricity∈0,1.

Concentricity closer to one implies a better concentric structure.

**Figure 10:**
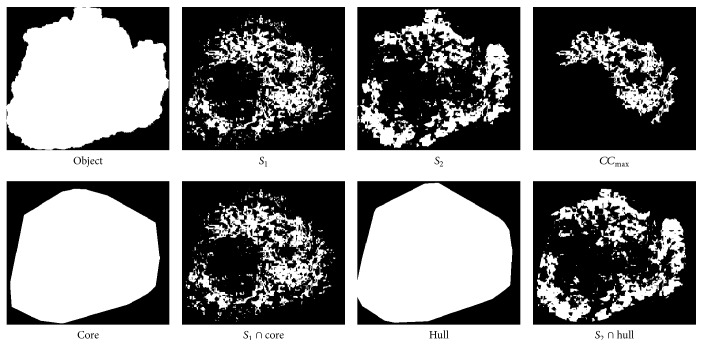
Regions used to compute concentricity of an object.

Images of all regions used to compute concentricity have been compiled in Figure 10.

The centroid distance for a color channel is defined as the distance between the geometric centroid (of the binary object) and the brightness centroid of that channel. The brightness centroid may be considered a center of mass of an object whose density is determined by intensity values of its pixels. If the pigmentation in a particular channel is homogeneous, the brightness centroid will be close to the geometric centroid resulting in small value of the centroidal distance.

Color similarity of two regions has been quantified by the *L*_1_- and *L*_2_-norm histogram distances for CIE *L* ^*∗*^ *a* ^*∗*^ *b* ^*∗*^ color space coarsely quantized into 4 × 8 × 8 bins [24]:

(10) L1HA,HB=∑i=14×8×8HAi−HBi,L2HA,HB=∑i=14×8×8HAi−HBi2,

where *H*_*A*_, *H*_*B*_ are histograms of areas *A* and *B*, respectively.

### 2.5. Texture Description

Quantitative properties of lesion's texture were described with measures based on a gray level cooccurrence matrix (GLCM) and a gray level run-length matrix (GLRLM) [28, 29].

GLCM is a square matrix *P*, where the (*i*, *j*)th entry of *P* represents information about the frequency of occurrence of such two adjacent pixels, where one of them has intensity *i* and another has intensity *j*. Such information may be used to derive second-order statistical measurements characterizing examined texture. GLCM and six measures derived from it (contrast, correlation, energy, homogeneity, maximum probability, and dissimilarity) have been calculated as described by Celebi et al. [24]:

(11) Contrast=∑i,ji−j2Pij,Correlation=−∑i,ji−μxj−μyσxσyPij,Energy=∑i ∑jPij2,Homogeneity=∑i ∑jPij1+i−j2,Maximum Probability=maxi,j⁡Pij,Dissimilarity=∑i ∑jPij·i−j.

With GLRLM, it is possible to analyze higher order statistical features for the given texture. GLRLM is a two-dimensional matrix in which each element *p*(*i*, *j*∣*θ*) gives the total number of occurrences of runs of length *j* at gray level *i*, in a given direction *θ*.

In our study, the direction of runs is irrelevant, as dermoscopic camera has no fixed orientation when taking images of lesions—there is no “reference orientation.” The only thing that matters is texture's homogeneity; thus instead of calculating measures for individual orientations *θ* ∈ {0°, 45°, 90°, 135°}, all GLRLM computed for the aforementioned orientations have been added together and measures have been computed only using this new orientation-invariant matrix $\hat{P}$.

GLRLM is used to derive 11 measures. Five basic measures (SRE, LRE, GLN, RLN, and RP) describe the distribution of runs' lengths (SRE, LRE, RLN, and RP) and runs' intensity (GLN) [29]. As SRE and LRE do not consider pixels' intensity, LGRE and HGRE measures have been proposed [30]. Finally, SRLGE, SRHGE, LRLGE, and LRHGE measures are based on statistical properties of a joint probability distribution of both runs' intensity and length [31]:

(12) SRE=1nr∑i=1G ∑j=1RP^ijj2,LRE=1nr∑i=1G ∑j=1Rj2P^ij,GLN=1nr∑i=1G∑j=1RP^ij2,RLN=1nr∑j=1R∑i=1GP^ij2,RP=nrn,LGRE=1nr∑i=1G ∑j=1RP^iji2,HGRE=1nr∑i=1G ∑j=1Ri2P^ij,SRLGE=1nr∑i=1G ∑j=1RP^iji2·j2,SRHGE=1nr∑i=1G ∑j=1Ri2P^ijj2,LRLGE=1nr∑i=1G ∑j=1Rj2P^iji2,LRHGE=1nr∑i=1G ∑j=1Ri2j2P^ij,

where *n* is the number of image pixels with *G* gray levels, *R* is the maximal run length, and *n*_*r*_ is the total number of runs in an image ($n_{r}=\sum_{i=1}^{G}\sum_{j=1}^{R}{\hat{P}}_{ij}$).

### 2.6. Asymmetry

The observed asymmetry is a significant diagnostic premise, as in malignant lesions the arrangement of local structures (e.g., dots, streaks, and pigmentation nets) is nonuniform across the whole area of a lesion [32]. Some researchers point out that, for instance, sharp transitions between central and border area indicate malignancy [24, 33].

Asymmetry measures may be defined in terms of ratios of feature values across various lesion's area segments or in terms of changes of feature values between halves obtained by splitting lesion area into halves using straight section lines passing through the center of mass [32, 33].

**Figure 11:**
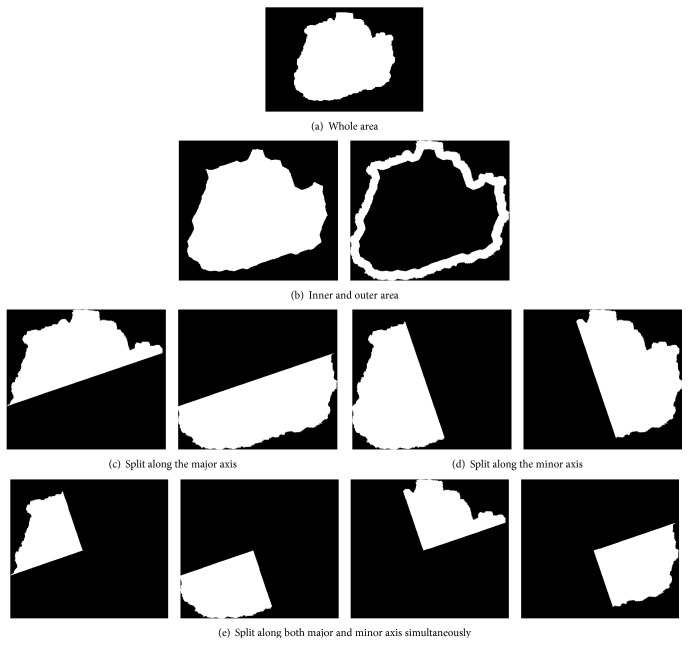
A sample division of an object into subregions.

In our study, a different approach has been adopted. The lesion area has been first divided into sets of subregions in three different manners (Figure 11) by splitting (1) into central and border part, (2) into halves along minor and major axis, and (3) into quarters using same axes. Then a variance of feature values has been calculated for each set of subregions. Additionally, asymmetry indices proposed by Stoecker et al. [34] and the following quantities have been computed:

(13) $$\begin{array}{c} R_{Q_{i}}=\frac{f_{Q_{i}}}{f_{W}}, \end{array}$$

where *f*_*Q*_*i*__ denotes value of feature *f* calculated for *i*th quadrant and *f*_*W*_ for the whole area, respectively.

### 2.7. Data Preparation

In order to apply correlated-base feature selection method (the choice of this particular method is discussed later), numerical features have been discretized. This is due to the fact that in the studied problem the decision feature is a categorical variable, which means it takes on discrete values, and the relationship between variables of those two classes cannot be directly assessed. The only solution is to discretize the variable taking continuous values. In general terms, discretization is a simple logical condition, which takes into account one or a few attributes and which aims at splitting a range of data into at least two subsets. Data in our study have been discretized according to the method proposed by Fayyad and Irani [35], which uses heuristics minimizing entropy based on the “minimum description length principle.”

To ensure correct work of algorithms based on analysis of distances between points in the feature space (e.g., *k*-nearest neighbors algorithm, SVM), for each feature its values have been scaled to the range of [−1; 1]. Consequently, the risk of a situation in which a feature with larger range of values dominates other features has been eliminated. Normally distributed features have been transformed using *Z*-score (Equation (14)), whereas other features were rescaled linearly to the desired range (15) [36]. Consider

(14) x^=x−x¯3swhere  x¯=1N∑ixi,  s=1N−1∑ixi−μ2,

(15) x^=2xi−xminxmax−xmin−1,

where **x** is a vector of feature's values prior to normalization and $\hat{x}$ is the same vector after normalization. To determine if a given feature is well modeled by a normal distribution, chi-square goodness-of-fit test has been applied.

### 2.8. Class Imbalance Problem

The data set used in this study exhibits class imbalance problem which means that the classes are not approximately equally represented (i.e., it consists of more than three times as many images of Clark nevus than images of blue nevus). In such case, most classifiers will focus on learning how to identify representatives of majority classes leading to poor predictive accuracy for minority classes. When making a diagnosis, such a situation is unacceptable, as misclassification error may lead even to patient's death.

The most common way to address this issue is sampling [51]. There exist two main types of sampling methods:* undersampling*, which consists in removing representatives of the majority class, and* oversampling*, which consists in adding examples of the minority class. Out of many sampling techniques, two methods are particularly popular:* random undersampling* and* synthetic minority oversampling technique* (SMOTE). In random undersampling method, randomly drawn representatives of the majority class are removed from the data set. In SMOTE method, new “synthetic” examples are created by averaging a few representatives of the same minority classes which are nearest neighbors in the feature space [52].

In this study, SMOTE method has been used, as it has been proven to be more effective than random undersampling when tested on a set of dermoscopic images obtained from various sources, without any common or rare lesion types omitted [24].

### 2.9. Feature Selection

Feature selection consists in reducing data dimensionality by rejecting redundant, unimportant, or noisy features, thus resulting in increased prediction accuracy, less complex classifier models, and better computation efficiency.

Feature selection algorithms may be divided into three main categories:* filters*,* wrappers*, and* projections* [53, 54]. In this study, filter methods have been used for two reasons [24]. Firstly, as filters are usually fast, it is possible to test a huge number of combinations of features. Secondly, if one would like to use wrappers on a given data set, the target learning algorithm should demonstrate satisfactory results for the original data set, as wrappers are based on feedback principle. As some features extracted in this study might be irrelevant or redundant as well as due to class imbalance, wrappers have not been likely to fulfill those restrictions. On the other hand, projections are used mainly to increase computational performance, which was not the case in this study.

Out of numerous available filters, three are worth noticing due to their satisfactory performance on various data sets [53]: ReliefF, mutual information-based feature selection, and correlation-based feature selection [55–57]. As numerous features extracted in this study are strongly mutually correlated, correlation-based feature selection has been chosen as the only method which takes into account not only relationships between features and the decision class, but also relationships between features themselves.

The heuristic “merit” Merit_*S*_ of a feature subset *S* containing *k* features is given by [58]

(16) $$\begin{array}{c} {Merit}_{S}=\frac{k\bar{r_{cf}}}{\sqrt{k+k\left(k-1\right)\bar{r_{ff}}}}, \end{array}$$

where $\bar{r_{cf}}$ is the average feature-class correlation and $\bar{r_{ff}}$ the average feature-feature intercorrelation.

To compare two feature vectors of discrete values, the* symmetric uncertainty*, an improved version of information gain measure, has been used [59]. Symmetric uncertainty is given by

(17) $$\begin{array}{c} \text{SU}\left(\;X,Y\;\right)=2\left[\;\frac{H\left(X\right)+H\left(Y\right)-H\left(X,Y\right)}{H\left(Y\right)+H\left(X\right)}\;\right], \end{array}$$

where *H*(*A*) is the marginal entropy of set *A* and *H*(*A*, *B*) is the joint entropy of sets *A* and *B*.

The number of selected features is a parameter which requires a careful tuning: too few features results may prevent classifiers from distinguishing between various classes whereas too many features impose risk of overfitting—a situation when a model excels in classifying training data but fails to generalize knowledge and hence misclassifies new samples. As some researchers suggest limiting the number of features, *k*, to the range of 5 ≤ *k* ≤ 30 [24], in this study *k* = 20 has been adopted.

The applied procedure allowed choosing features which best discriminate lesion types: concentricity computed using *k*-means algorithm, concentricity computed using kernel *k*-means algorithm for *σ* = 1, *L* ^*∗*^ *a* ^*∗*^ *b* ^*∗*^ histogram distances in *L*_1_ and *L*_2_ metrics computed for central and border part, variances from sets of *L* ^*∗*^ *a* ^*∗*^ *b* ^*∗*^ histogram distances between the whole region and individual quarters in both *L*_1_ and *L*_2_ metrics, variances from sets of *L* ^*∗*^ *a* ^*∗*^ *b* ^*∗*^ histogram distances between each pair of quarters in both *L*_1_ and *L*_2_ metrics, variances from sets of GLCM features (dissimilarity, maximum probability, entropy, energy, correlation, and contrast) computed for quarters, variance of variances of *a* ^*∗*^ channel values (in *L* ^*∗*^ *a* ^*∗*^ *b* ^*∗*^ color space) calculated for quarters, variance of variances of H channel values (in HSV color space) calculated for quarters, mean value of variances of *a* ^*∗*^ channel values (in *L* ^*∗*^ *a* ^*∗*^ *b* ^*∗*^ color space) for quarters, variance from sets of *L* ^*∗*^ *a* ^*∗*^ *b* ^*∗*^ histogram distances between halves in both *L*_1_ and *L*_2_ metrics, and variance from sets of GLCM dissimilarity computed for halves.

This particular choice of features does not diverge from the clinical practice, according to which asymmetry is one of the most important premises in determining the lesion type.

### 2.10. Classification

#### 2.10.1. Models

The following predictive models have been tested [60, 61]: *k*-nearest neighbors algorithm (for *k* ∈ {3,5, 10,15}), logistic regression, decision tree, and support vector machine (SVM). Other models, like neural networks or rule-based systems, have not been a subject of this study [62]. In case of the decision tree, Gini-Simpson index has been used as a measure of data diversity [63]. Decision boundary of SVMs has been determined using radial basis function as kernels. Radial basis function has been preferred over linear, sigmoid, and polynomial kernels as it exhibits a few important properties [24]: it allows classifying nonlinearly separable data sets (as is the case in this study), it is characterized by high numerical stability, and it is defined using only one parameter. Moreover, as in this study there are as many as four decision classes, the multiclass classification problem has been decomposed into a (greater) number of binary classification problems. Winner-takes-all strategy has been applied to combine solutions of subproblems into a final solution, as for small data sets its results are similar to the results of the best strategy—pairwise coupling—while at the same time its computational burden is much lower [64, 65].

Individual classifiers have been trained using typical parameters. It has been assumed that the cost of misclassifying melanoma as nevi is four times higher than another sort of misclassification. Initial experimental results have proven SVM to be the most effective classifier (similar observations have been made by other research teams [60, 66]). Consequently, further experiments were focused on improving performance of SVMs by fine-tuning their parameters.

SVM with radial basis function kernel is governed by two parameters—misclassification cost, *C*, and kernel width, *γ*. The task is to select those parameters in such a way that the accuracy of predictions for new data (data not used in the learning process) is maximal.

As values of only two parameters have had to be adjusted, grid search has been applied [67]. For each parameter, values from exponential series have been considered: *C* ∈ {2^−5^, 2^−3^,…, 2^15^} and *γ* ∈ {2^−15^, 2^−13^,…, 2^3^}. Each combination of parameter values (*C*_0_, *γ*_0_) has been assessed using a validation procedure described below. After the grid search had finished, SVM have been trained using optimal parameter values (*C* ^*∗*^, *γ* ^*∗*^).

#### 2.10.2. Validation

The aim of the validation step is to assess classifier's quality based on the number of generalization errors, that is, misclassified samples. Optimal SVM models have been validated using stratified Monte Carlo cross validation method and in each iteration the test set consisted of 10% of samples.

The Monte Carlo variant of cross validation had been chosen for a number of reasons. Firstly, experimental results obtained for a similar problem [24] suggest high effectiveness of this method. Secondly, in their analysis, Molinaro et al. [68] point out that, for datasets consisting of few samples with many features, such as a dataset used in this study, the difference in effectiveness between Monte Carlo and “classic” 10-fold cross validation is insignificant. The number of samples drawn into the test set has been determined using a “rule of thumb” [69]. Finally, by applying Monte Carlo cross validation, one may avoid constructing overdeveloped predictive models, which decreases the risk of overfitting.

As in the data set used in this study, there is a considerable difference in the number of representatives between various decision classes and stratification has been applied. Stratification ensures the similar distribution of representatives of each decision class in both training set and test set in each iteration of the validation procedure.

## 3. Results and Discussion

### 3.1. Database Specification

The described algorithm for the automatic classification of specific melanocytic lesions has been tested on dermoscopic images from a widely used Interactive Atlas of Dermoscopy [2]. Images for this atlas have been provided by two university hospitals (University of Naples, Italy, and University of Graz, Austria) and stored on a CD-ROM in the JPEG format. The documentation of each dermoscopic image was performed using a Dermaphot apparatus (Heine, Optotechnik, Herrsching, Germany) and a photo camera (Nikon F3) mounted on a stereomicroscope (Wild M650, Heerbrugg AG, Switzerland) in order to produce digitized ELM images of skin lesions. All the images have been assessed manually by a dermoscopic expert with an extensive clinical experience.

Furthermore, all the descriptions of skin cases were based on the histopathological examination of the biopsy material. In order to develop and test the automatic procedure for the classification of melanocytic skin lesions, 300 images with different resolutions, ranging from 0.033 to 0.5 mm/pixel, were chosen. The database included 100 Clark nevus cases, 70 blue nevus cases, 70 Spitz nevus cases, and 60 malignant melanomas.

The preprocessing step (black frame removal and hair removal) as well as the segmentation step (border error less than 6%) did not affect the further research [14].

### 3.2. Statistical Analysis

The performance of a classifier can be assessed based on the analysis of discrepancies in classification, that is, differences between the classification carried out by the classifier and the actual classification (ground truth), summarized in a confusion matrix. In this matrix, each row refers to actual classes, *c*(*x*), as recorded in the test set, and each column refers to classes as predicted by the classifier, $\hat{c}(x)$. The (*i*, *j*)th element contains the number of test instances predicted by a classifier to belong to *j*th class class, whereas they are actually representatives of *i*th class.

From a contingency table we can calculate three performance indicators: accuracy, true positive rate (TPR), and true negative rate (TNR).

For a binary classification problem, those indicators are given by [70]

(18) Accuracy=1Te∑x∈TeIc^x=cx,TPR=∑x∈TeIc^x=cx=⊕∑x∈TeIcx=⊕,TNR=∑x∈TeIc^x=cx=⊖∑x∈TeIcx=⊖,

where Te is the test set and the function *I*[·] denotes the indicator function. Positive and negative classes are denoted by ⊕ and ⊖, respectively.

Although the initial problem is a multiclass classification problem (with four classes), it can be decomposed into four binary classification problems: whether an instance belongs to the given class or not.

When dealing with a data set exhibiting class imbalance problem, the accuracy should be considered only a preliminary performance indicator. For instance, for a data set with class ratio 99 : 1, a classifier which simply counts each instance as a representative of the majority class would score the accuracy of 99%! Therefore in such cases better measure would be a plot of the ROC curve and the area under that curve.

The ROC plot completely visualizes the (normalized) contingency table, by means of plot in the unit square with TPR rate on the *y*-axis and TNR rate on the *x*-axis. Each of the points marked on the ROC plot specifies the classification performance, in terms of true and false positive rates, achieved by the corresponding score thresholds. In our study, posterior probabilities have been used as scores. Those points are connected by straight lines producing a piecewise linear curve, the ROC curve, that rises monotonically from (0,0) to (1,1). The ROC plot can be interpreted as a plot of costs-to-benefits ratio. To measure the performance of a classifier using a ROC plot, the area under that plot curve (AUC) is calculated.

**Figure 12:**
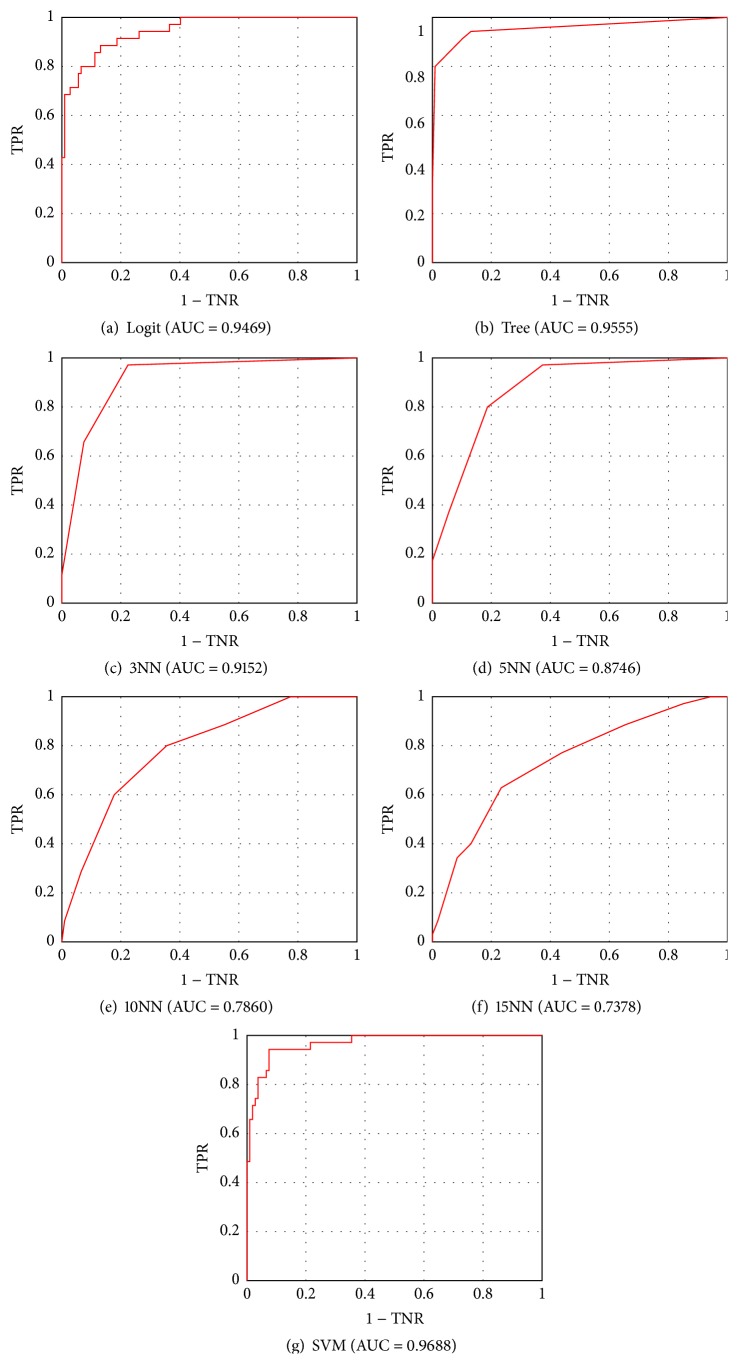
ROC plots for individual classifiers for a malignant-or-benign classification.

Values of aforementioned performance indicators for all examined classifiers have been summarized in Table 2. Additionally, Figure 12 presents ROC plots for a malignant-or-benign classification.

Among all examined classifiers, SVM achieved definitely best results. It achieved highest overall accuracy ACC_ALL_ = 0.9296, whereas, for the second-best classifier, the logistic regression, ACC_ALL_ = 0.8028. The SVM outperformed the logistic regression in classifying all four lesion types. It is to be stressed that the SVM turned out to be the most effective classifier in recognizing malignant melanoma, the most dangerous of all four lesions. Its true positive rate and area under the curve amounted to TPR_MM_ = 0.8611 and AUC_MM_ = 0.9688, respectively. A little bit lower true negative rate for the SVM than for the decision tree is not a problem in the studied area. It indicates misclassification of Clark or Spitz nevi as malignant melanoma, and the cost of such a misdiagnosis is much, much lower than of classifying a malignant tumor as a benign one.

The achieved results are slightly better than most results of malignant-or-benign studies conducted so far. Our method allowed classifying malignant lesions with TPR_MM_ = 0.8611 and TNR_MM_ = 0.9623, whereas for other similar studies TPR falls within the range 0.8–1.0 and TNR –0.5–0.95, respectively [66, 71].

A truly reliable diagnosis can be made only by a histopathological examination of a lesion. When in their studies Curley et al. asked three experienced dermatologists to classify lesions based only on the visual examination, those physicians identified correctly only half of cases. Therefore, the presented method of classification yields better results than those obtained during a medical examination.

## 4. Conclusions

This paper presents a computer-aided approach for the automatic classification of melanocytic lesions and accurate diagnosis of melanoma. In our research we evaluated our approach with four types of classifiers: *k*-nearest neighbors algorithm, logistic regression, decision tree, and SVM. The developed system achieved 92% accuracy with SVM.

Our study gives an important contribution to the research area of skin lesion classification for several reasons. Firstly, it focuses on specifying the exact type of a skin lesion and not only on categorization of skin lesions as benign or malignant. Secondly, this work combines the results of research done so far related to all the steps needed for the development of an automatic diagnostic system for melanocytic lesion detection and classification. Finally, the refinement of current approaches and development of new techniques and methods will help to improve the ability to diagnose skin moles more precisely and to achieve the goal of the significant reduction in melanoma mortality rate.

### 4.1. Future Work

Starting from the present framework, further research efforts will be firstly addressed to compare and integrate the very promising approaches and corresponding feature descriptors reported in the most recent literature, in order to improve the classification accuracy of melanocytic lesions. We will also conduct a follow-up study by collecting more real data, especially melanoma cases, to further evaluate our approach. One of the major problems in the field of melanocytic skin tumors is the underdiagnosis of melanoma as a benign melanocytic or nonmelanocytic lesion. To decrease the amount of dermoscopic pitfalls, the number of melanocytic lesion types will be extended for a better evaluation of melanocytic lesion.

## Figures and Tables

**Table 1:** A categorization of feature descriptors commonly used in the computerized analysis of dermoscopic images.

Clinical features	Feature descriptors	References
Asymmetry	Symmetry distance	[37]
	Lesion's centroid	[38]

Border irregularity	Fourier feature	[39]
	Fractal geometry	[40]
	Area and perimeter	[38, 41, 42]
	Irregularity index	[43, 44]

Color variegation	RGB statistical descriptors	[38, 45]

Diameter	Semimajor axis of the ellipse	[38]

Other features	Pattern analysis	[38, 46, 47]
	Wavelet-based descriptors	[48]
	Texture descriptors	[49]
	Intensity distribution descriptors	[5]
	Haralick descriptors	[50]

**Table 2:** The assessment of classifiers for a 1-vs-all and overall classification.

Measure	Logit	Tree	3NN	5NN	10NN	15NN	SVM
TPR_BN_	0.9730	0.8485	0.7200	0.7660	0.6939	0.6735	1.0000
TNR_BN_	1.0000	0.9266	1.0000	1.0000	0.9785	0.9677	1.0000
AUC_BN_	1.0000	0.9675	0.9914	0.9889	0.9679	0.9659	1.0000

TPR_CN_	0.8611	0.6977	0.8519	0.7586	0.8148	0.8182	0.9143
TNR_CN_	0.9528	0.9394	0.8870	0.8761	0.8783	0.8500	0.9626
AUC_CN_	0.9817	0.9279	0.9615	0.9089	0.9099	0.9030	0.9851

TPR_MM_	0.8387	0.9655	0.7419	0.6786	0.6071	0.5185	0.8611
TNR_MM_	0.9189	0.9381	0.8919	0.8596	0.8421	0.8174	0.9623
AUC_MM_	0.9469	0.9555	0.9152	0.8746	0.7860	0.7378	0.9688

TPR_SN_	0.8421	0.7568	0.8235	0.7105	0.6053	0.5227	0.9429
TNR_SN_	0.9712	0.9333	0.9352	0.9231	0.8846	0.8776	0.9813
AUC_SN_	0.9648	0.9425	0.9606	0.9395	0.8812	0.8545	0.9789

ACC_ALL_	0.8803	0.8028	0.7746	0.7324	0.6761	0.6197	0.9296

BN: blue nevus, CN: Clark nevus, MM: malignant melanoma, SN: Spitz nevus, ALL: multiclass classification.
